# Exhaustive valorization of cashew nut shell waste as a potential bioresource material

**DOI:** 10.1038/s41598-021-91571-y

**Published:** 2021-06-07

**Authors:** James Nyirenda, Kadango Zombe, George Kalaba, Chipo Siabbamba, Inyambo Mukela

**Affiliations:** 1 Department of Chemistry, School of Natural Sciences, The University of Zambia, Box 32379, Lusaka, Zambia; 2 Zambia Agriculture Research Institute, Mongu, Zambia

**Keywords:** Chemical biology, Environmental sciences, Environmental social sciences, Chemistry, Materials science

## Abstract

In this paper, we report extraction of cashew nut shell liquid (CNSL) from cashew nut shell waste (CNSW) and further use of residues for generation of activated carbon for removal of heavy metals and methylene blue (MB). Solvent extraction yielded 24.6 ± 0.4%, 38.2 ± 0.4% and 40.1 ± 0.9% for petroleum ether, hexane and ethanol respectively. Phytochemical screening showed presence of alkaloids, carbohydrates, saponins, phenols, tannins, flavonoids, amino acids, terpenoids, proteins, steroids, glycosides and carboxylic acids. The CNSL had a pH of 3.2, viscosity (104.6 ± 1.8 mPa s), moisture (6.5%), ash (1.6 ± 0.1%), refractive index (1.52 ± 0.001), specific density (0.9561 ± 0.0002 g/cm^3^), acid value (118.7 ± 9.2 mg KOH/g), free fatty acid value (60.1 ± 4.7%), saponification number (138.1 ± 3.2 mg KOH/g) and iodine value (188.1 ± 2.3 mgI 2/100 g). The average percentage removal of Cu (II), Pb (II), Cd (II) and Zn (II) was 99.4 ± 0.5, 95.4 ± 1.5, 99.5 ± 0.1, 98.4 ± 0.1%, and removal efficiency of MB at 50, 150, 250 and 350 mg/L was 99.63, 97.66, 96.48 and 94.81%, respectively. Equilibrium data were best described by the Freundlich isotherm model. The maximum monolayer adsorption capacity was 12.1 mg/g. The adsorption kinetics conformed to pseudo-second-order model. ∆G° was negative and a ∆H° of + 22.76 kJ/mol indicated that adsorption was endothermic. The ΔS° (+ 0.086 kJ/mol/K) showed that there was spontaneous interaction of the solution and adsorbate. These results show that CNSW is a potential bioresource for CNSL production for use in the paints, varnishes, surface coatings, agrochemicals and ethnomedicine industries. Residual shells can be exploited as fuels or converted to activated carbon for use as low-cost filters in water purification.

## Introduction

The cashew tree (*Anarcardium occidentale*) is a native of Brazil and the Lower Amazons. The major producing countries of cashew are Tanzania, India, Mozambique, Sri lanka, Kenya, Madagascar, Thailand, Malaysia, Indonesia, Nigeria, Senegal, Malawi and Angola^1^. In Zambia, Cashew trees were first introduced in 1940s by the Portuguese traders in Western Province (then, Barotseland), an area characterized by Kalahari sandy soils that are relatively poor for most conventional crops. In order to diversify the economic base of the poor households in the Western Province, the Government of the Republic of Zambia (GRZ) promoted the planting and processing of cashew trees in the 1980s. However, the growth of the cashew industry was very slow due to low production and lack of marketing and processing facilities^2^.

In 2015 Zambia acquired a loan from Africa Development Bank under the Cashew Infrastructure Development Project (CIDP) to boost cashew nut production^2,3^ as well as increasing production in the province that has largely remained poor for a long time^4^. In Zambia, cashew is grown mainly for its kernels while the shells are discarded in the environment as waste hence, contributing to the already existing waste management crisis in the country^5^. However, unlike other solid wastes, the cashew nut shell (CNS) harbour’s a dark brown viscous oil called cashew nut shell liquid (CNSL), located in the soft honeycomb structure found between the inner and outer shell^6^. Cashew nut shell liquid contributes approximately 30–35% to the total weight of shell and is by far the most important constitute of the shell^7,8^. It is a cheap, abundantly available, and renewable raw material with diverse industrial applications and biological activities^9,10^. It is composed of four naturally occurring substituted phenols that have great potential to replace synthetic phenols in many applications with equivalent or better results^9^. The extraction of CNSL from the CNS involves three major methods and depending on the method employed, CNSL can be classified into two types: technical and natural^11^. Natural CNSL is extracted by solvent extraction and mechanical pressing of the shell. It contains anacardic acid (70%), cardol (18%), cardanol (5%) and traces of methyl cardol^8^. It is best known for its diverse biological activities, with anacardic acid been linked to the observed physiological effects^12^. Among them being antimicrobial, fungicidal, insecticidal, termiticidal, antioxidant and enzymatic inhibition properties^13–18^. Technical CNSL is obtained by heating natural CNSL at temperatures above 180–200 °C. During the heat process, the thermolabile anacardic acid decarboxylate and converts to cardanol, leading to high content of cardanol (60–65%) in technical CNSL^19^. This form of CNSL has found wide industrial applications as raw material in friction linings, paints, varnishes, laminating, epoxy resins, foundry chemicals, plastic formulations and as antioxidant in biodiesel^17,20,21^. The innumerable industrial applications of CNSL are based on the fact that it leads itself to polymerization by various means. Simple phenols from petrochemicals have restrictions hence, the range of products obtained from them are few^1^. The current rise in the prices of petrochemical feedstocks as well as concerns of environmental pollution and depletion of natural reserves, puts CNSL at the center stage as the best sustainable alternative source of renewable energy^9^. Its advantages surpass those of other competing renewable bioresources such as vegetable and corn oils. Cashew nut shell liquid is non-edible hence, it does not put pressure on the food supply chain, and the fact that it is sourced from waste raw materials, it does not compete for production land^9,22^. Other parts of the *Anarcardium occidentale* plant have been exploited for their medicinal values^23,24^. The fruit juice and the nut shell oil are both said to be folk remedies for cancerous ulcers, elephantiasis and warts. The oily substance from pericarp is used for cracks on the feet^25^. Old leaves are applied to skin afflictions and burns. In Ghana, the bark and leaves are used for sore gums and toothache. Decoction of the astringent bark is suggested for severe diarrhea and thrush. In India, bark is used in herbal tea for asthma, cold and congestion and as an antidote for snake bites^26^. Other uses of cashew nut shell liquid derivatives include anticancer and cardiovascular activity^14,,18,27^. In additional, residual shells after extraction of CNSL can be exploited as a source of fuel^28^, or as gasifier feed stocks^29^, or can be convert to bio-filters to remove heavy metals and organic pollutants from waste waters through adsorption processes^30^. The nature of CNS would make synthesis of bio-filters very cheap and accessible to the locals. The increase in chemical industries, agricultural activities and abuse of water resources has contributed greatly to water contamination by heavy metals and organic compounds such as paints, dyes, waste chemical effluents and agrochemical residues. A study by Ikenaka et al.^31^ showed that heavy metal pollution in many parts of Zambia includes high copper, zinc, cadmium and lead concentrations. Accumulation of these metals in the human body can cause carcinogenesis, neurotoxicity, cell damage and loss of cellular functions^32^. The maximum permissible limit of cadmium, zinc, copper and lead in drinking water by World Health Organization are 0.003 mg/L, 3 mg/L, 2 mg/L and 0.01 mg/L respectively^33^. Therefore, removal of these heavy metals from drinking water is a priority. In other studies^34^, pharmaceutical products have been shown to have low biodegradability and hence find themselves in wastewater and surface waters^35^. Other studies have shown potential of contamination of water and soil by agricultural chemicals such as pesticides^36^. The conventional techniques for removal of waste from water includes; ion exchange process, chemical precipitation, membrane separation, ultra-filtration, chemical oxidation, reverse osmosis process and many others. These techniques are costly and requires high energy input. On the other hand, adsorption has a greater advantage because it is simple, safe and less costly^37^. The main objective of this research was to extract CNSL, determine its phytochemical composition and physicochemical properties, as well as designing a low-cost adsorbent material from defatted shells which can be used to remove heavy metals and organic pollutants from water. Various literature cited gave this research room for adding baseline data for the Zambian grown cashew, as the family Anacardiaceae covers over 70 genera in which more than 600 species are distributed in tropical, sub-tropical and temperate regions in the world^38^ hence the plant cannot be easily generalized that Zambia has only one family or subspecies. Hence this paper seeks to evaluate the value of cashew nut shell waste for potential valorisation, wealth and employment creation under small scale enterprises.

## Materials and methods

### Materials

Concentrated sulfuric acid (H_2_SO_4_), hydrochloric acid (HCl) and nitric acid (HNO_3_) (purchased from Hi-Media) were a kind donation by Medical Stores Limited (Ministry of Health, Zambia). Analytical grade Mercuric chloride (HgCl_2_), sodium carbonate (Na_2_CO_3_), sodium citrate (NaC_6_H_7_O_7_), metallic magnesium (Mg), lead acetate (C_4_H_12_O_7_Pb), sodium hydroxide (NaOH) and methylene blue (C_16_H_18_CIN_3_S) (BDH) were all supplied by the Department of Chemistry, University of Zambia.

Cadmium nitrate tetra-hydrate (Cd (NO_3_)_2_·4H_2_O), Zinc nitrate hexa-hydrate (Zn (NO_3_)_2_·6H_2_O), Copper (II) sulphate (CuSO_4_·5H_2_O), lead (II) nitrate (Pb (NO_3_)_2_), hexane (C_6_H_12_), ethanol (C_2_H_5_OH), petroleum ether (40–60 °C), chloroform (CHCl_3_), acetic anhydride ((CH_3_CO)_2_O), methanol (CH_3_OH), acetic acid (CH_3_COOH), ninhydrin, potassium iodide (KI), potassium iodate (KIO_3_), Sodium thiosulphate (Na_2_S_2_O_4_), ferric chloride (FeCl_3_), potassium sodium tartrate ( KNaC_4_H_4_O_6_·4H_2_O), picric acid (C_6_H_3_N_3_O_7_), α-naphthol (C_10_H_7_OH), were purchased from Sigma Aldrich under Merck.

### Methods

#### Sample preparation

Pre-processed (roasted) CNS were collected from small-scale cashew nut processors in Mongu District of the Western Province of Zambia. Upon their arrival in the laboratory, the shells were washed several times with tap water and twice with enough distilled water to remove all the dirty, contaminants and debris. After washing, the shells were air-dried under the shade for 7 days. Once dry, the shells were ground to homogeneity using a Thomas-Model-4-Wiley-Mill fitted with a 2 mm sieve, placed in airtight bags and stored in a refrigerator at 4 °C to avoid biological and chemical degradation of the constituents.

#### Extraction of cashew nut shell liquid

The extraction of CNSL from CNSW was carried out by using a soxhlet extractor system as described by^6,39^. Briefly, 20.00 g of ground CNSW was put into a clean 33 × 100 mm cellulose thimble (Whatman) and extracted with a particular solvent for 8 h. After several cycles of extraction, the soxhlet apparatus was disassembled and the remaining solvent in the extracting chamber was added to the other extract in round bottomed flask, and evaporated under mild conditions with a Buchi Rotavapor until a constant oily mass remained.

#### Phytochemical screening

Twenty grams of ground CNS were exhaustively extracted under cold conditions in 200 mL acetone, ethanol and hexane respectively for 72 h with interval shaking in the dark. The organic solvents were recovered under mild pressure with a Buchi Rotavapor. The effect of solvent on phytochemical is presented in Table 1. The water extract was warmed at 60 °C for 10 min and left to stand for a total of 24 h with interval shaking. Phytochemical analysis for alkaloids, flavonoids, glycosides, phenols, saponins, steroids, tannins and terpenoids was done according to Refs.^40–45^ and for amino acids, carbohydrates, carboxylic acids and proteins^46^ with minor modifications. All reagents used in this process were prepared fresh before use.

**Table 1:** Phytochemical Screening results for CNSW extracted with different solvents.

Phytochemical compounds	Aqueous	Ethanol	Acetone	Hexane
Carbohydrates	++	+	+	+
Proteins	−	+	+	++
Amino acids	++	−	−	+
Phenols/tannins	++	+++	+++	+++
Alkaloids	** − **	+	+	+
Saponins	+	++	++	++
Flavonoids	++	++	++	++
Steroids	+	+	+	+
Terpenoids	−	+	+	+
Glycosides	−	+	−	+
Carboxylic acid	+	+	+	+

Table Shows the phytochemicals present in various solvent types. Hexane was chosen as a solvent of choice due to extraction of all tested secondary metabolites.

#### Physicochemical characterization of cashew nut shell liquid

Methods by Refs.^1,11,47^ with minor modifications were followed for characterization of the CNSL extracted from the roasted CNS. Moisture content was determined by heating 2.0 g of sample to a constant weight in a crucible placed in a Memmert oven (Memmert GmbH + Co. KG) maintained at 105 °C for 3.5 h. The crucible was cooled in the desiccator and reweighed, the mass change in the sample was recorded. Ash was determined by incinerating 1.0 g sample in a Carbolite muffle furnace (HTF ELP4, Bamford, Sheffield UK) maintained at 550 °C for 5 h^1^ using sintered glass crucibles. Specific gravity was determined using a standard pycnometer bottle with a stopper. The 25 mL bottle was filled with distilled water and the CNSL respectively and weighed independently. The acid and free fatty acid values were determined using the methods of Refs.^48–50^. The saponification number and iodine values were determined by the method of Ref.^51^. Refractive index at 20 °C was determined using Bellingham Stanley Abbe refractometer. Viscosity was determined by the Oswald viscometer using distilled water as a reference at 24 °C. The pH was determined with a calibrated pH meter (Crison base 20).

#### Preparation of activated carbon adsorbent

The defatted cashew nut shells were pre-heated at 110 °C for 2 h using a Carbolite AAF 11/7 Furnace at a heating rate of 10 °C/min. Chemical activation with 50 wt% sulphuric acid was carried out using an impregnation method. The impregnation ratio of sulphuric acid to the raw materials was 2: 1. Thus, 60 g of the pre-heated precursors were soaked in 86 mL of 50 wt% sulphuric acid for 24 h. After soaking, the precursor was dried in an oven at 110 °C. The dried precursors were carbonized in the same furnace as before at 400 °C for 3 h at a heating rate of 10 °C/min. The carbonized material was cooled to room temperature and washed severally with hot distilled water until the pH was neutral. The cooled activated carbon was then dried in an oven for 4 h, ground and sieved using a 0.5 mm sieve and stored in airtight bottles until use.

#### Analysis of heavy metals and methylene blue (MB)

Heavy metal concentrations were measured on a PerkinElmer Analyst 400 Atomic absorption spectrophotometer and a Shimadzu UV-2600 spectrometer was used to determine the concentration of Methylene blue standard before and after adsorption.

#### Adsorption experiments

Adsorption percentage (%) and the amount of adsorbate per unit mass of activated carbon (q_e_)^52,53^ was calculated using Eqs. (1) and (2).

1 $${\text{\% Adsorption}} = { }\frac{{\left( {{\text{C}}_{{\text{o}}} - {\text{C}}_{{\text{e}}} } \right) \times 100}}{{{\text{C}}_{{\text{o}}} }},$$

2 $${\text{q}}_{{\text{e}}} = \frac{{({\text{C}}_{{\text{o}}} - {\text{C}}_{{\text{e}}} ) \times {\text{V}}}}{{\text{m}}},$$

where; C_o_ is the initial concentration of adsorbate (mg/L), C_e_ is the final concentration of adsorbate after adsorption (mg/L), q_e_ is the amount of adsorbate adsorbed at equilibrium (mg/g), m is the mass of activated carbon used (g) and V is the volume of adsorbate solution used (mL).

#### Adsorption isotherm models

Three commonly models used to fit adsorption experiment results are the Langmuir, Freundlich and Temkin adsorption isotherm models^53,54^.

#### The Langmuir isotherm model

The isotherm assumes that the monolayer adsorption process happens between the adsorbate and homogenous surface of the adsorbent^55^. The binding sites have the same affinity for adsorption. The linear equation is given below;

3 $$\frac{{{\text{C}}_{{\text{e}}} }}{{{\text{q}}_{{\text{e}}} }} = \frac{1}{{{\text{K}}_{{\text{L}}} {\text{q}}_{{{\text{max}}}} }} + \frac{{{\text{C}}_{{\text{e}}} }}{{{\text{q}}_{{{\text{max}}}} }},$$

where; q_e_ is the metal ions adsorbed (mg/g) at equilibrium, C_e_ is the equilibrium concentration (mg/L), q_max_ is the monolayer adsorption capacity (mg/g) and K_L_ is the Langmuir adsorption constant which is related to the energy of adsorption and is a measure of the metal ions affinity to the adsorption sites. If the magnitude of K_L_ is large, the interaction of the adsorbent with the adsorbate molecules will be more while a smaller value indicates a weak interaction. The Langmuir parameters q_max_ and K_L_ were calculated from the slope (1/q_max_) and intercept (1/q_max_K_L_) of the plot of C_e_/q_e_ versus C_e._ An important characteristic of the Langmuir isotherm can be expressed in terms of the dimensionless equilibrium parameter or the separation factor, R_L,_ which is defined as;

4 $${\text{R}}_{{\text{L}}} = \frac{1}{{1 + {\text{K}}_{{\text{L}}} {\text{C}}_{{\text{o}}} }},$$

where; K_L_ is the Langmuir adsorption constant and C_o_ is the initial metal ion concentration. The value of the separation factor gives an indication of the shape of the isotherm and the nature of the adsorption process. The values of the R_L_ between 0 and 1 indicates favourable adsorption, unfavourable adsorption occurs when R_L_ is greater than 1 and adsorption is linear when R_L_ is equal to 1^56^.

#### The Freundlich isotherm model

The Freundlich isotherm model is an empirical model that explains that adsorption occurs on an unevenly distributed or heterogeneous surface of the adsorbent. The adsorbent surface has different affinity and energy for adsorption. Stronger binding sites are occupied first and then the binding strength decreases with the rise in the degree of site occupation. It is represented by the equation below;

5 $${\text{log q}}_{{\text{e}}} = \frac{1}{{\text{n}}}({\text{log C}}_{{\text{e}}} ) + {\text{log K}}_{{\text{F}}} ,$$

where; q_e_ is the metal ions adsorbed at equilibrium (mg/g), C_e_ is the equilibrium concentration (mg/L), and K_F_ is the Freundlich constant and n is the adsorption intensity. The value of n indicates the degree of non-linearity between metal ions concentration and its adsorption in the following manner; if n is equal to 1 (n = 1) then adsorption is linear, adsorption becomes a favourable physical process when n is greater than 1 (n > 1) and when n is less than 1 (n < 1) then adsorption is a chemical process^52,54^. From the slope (1/n) and intercept (log K_F_) of the plot of log q_e_ versus log C_e_, the constant K_F_ and n can be calculated.

#### Temkin isotherm model

The Temkin isotherm model considers the effect of indirect adsorbate-adsorbent interaction on the adsorption process. It is based on the assumption that the heat of adsorption of all the molecules in a layer decreases linearly due to increase in surface coverage of the adsorbent. The decrease in heat of adsorption is linear rather than logarithmic, as implied in the Freundlich isotherm. Further, the adsorption is characterized by uniform distribution of binding energies, up to a maximum binding energy. The Temkin isotherm model is represented by the following equation^53^:

6 $${\text{q}}_{{\text{e}}} = \frac{{{\text{RT}}}}{{\text{b}}}{\text{InK}}_{{\text{T}}} + \frac{{{\text{RT}}}}{{\text{b}}}{\text{InC}}_{{\text{e}}} ,$$

where; K_T_ is the equilibrium binding constant (L/mol) corresponding to the maximum binding energy, b is related to the adsorption heat, R is the universal gas constant (8.314 J/K/mol) and T is the temperature at 298 K. The constants K_T_ and b can be calculated from the slope (RT/b) and intercept (RTIn K_T_/b) of the plot of q_e_ versus ln (C_e_)^53^.

### Ethics approval and consent to participate

Ethical approval and waiver was obtained from the Natural and Applied Sciences Research Ethics Committee (NASREC) of the University of Zambia (UNZA) under the project REF. NO. NASREC: 2019-AUG-009.

## Results and discussion

**Figure 1:**
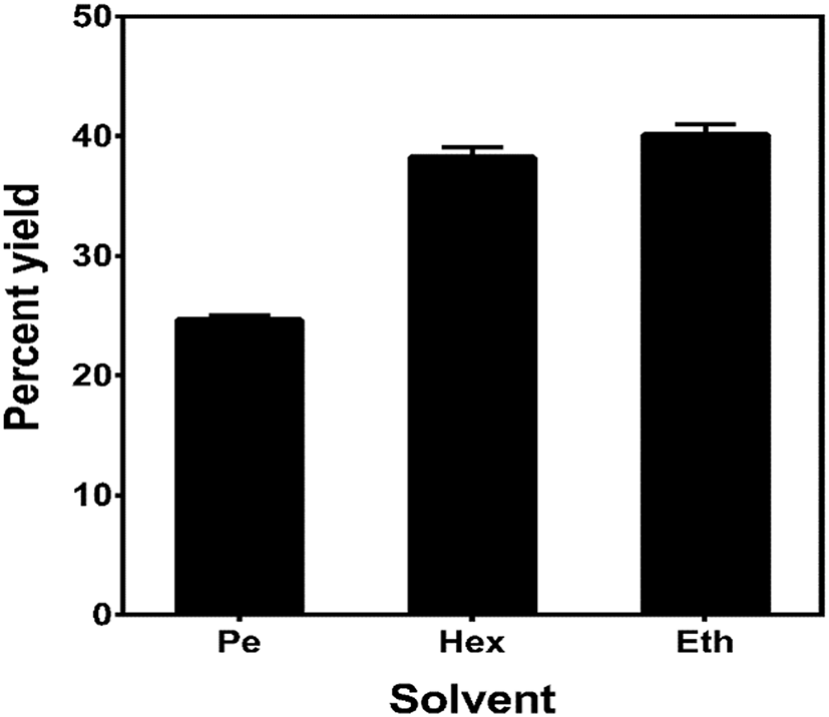
Effect of solvent on the yield of crude fat (CNSL) extraction from roasted cashew nut waste shell. The figure shows the amounts of crude fat in different solvents. The data is expressed as percent mean ± SEM. *Pe* petroleum ether, *Hex* hexane, *Eth* ethanol respectively.

Cashew nut shell liquid was extracted from CNSW with different organic solvents using a soxhlet extractor system. The percent yields are represented in Fig. 1. Extraction methods involving organic solvents under mild conditions have been reported to preserve the natural composition of CNSL^6^. The quality and percent yields of extracted CNSL varied among the organic solvents. The hight yield was recorded from ethanol 40.1 ± 0.9% followed by hexane 38.2 ± 0.4% and petroleum ether 24.6 ± 0.4% respectively. Although, ethanol recorded the highest yield, the quality of CNSL it extracted was poor, as it extracted more of undesirable polar coloured compounds from the shell^57^. Even during the carbonization process, ethanol defatted shells produced toxic fumes, which led to a conclusion that it did not completely remove CNSL from the shell^58^. Phytochemical analysis of the aqueous, ethanol, acetone, and hexane extracts of CNSW (Table 1) revealed the presence of phenols, tannins, flavonoids, saponins, steroids, terpenoids, glycosides, carboxylic acids, carbohydrates, proteins and amino acids. Various extracts from CNSW have been reported to have antimicrobial, antifungal^59^, insecticidal (Acero, 2018) and antioxidant properties^60^. Phyto-compounds such as saponins have been reported to have anticancer and anticholesterol activity^61^. Flavonoids and other polyphenolic acids show antioxidant, anti-inflammatory, antidiabetic and anticarcinogenic activities^62,63^. Alkaloids are natural anticancer and analgesic agents^64,65^. Steroids and terpenoids have anti-tumor, neuroprotection, antihypertensive, antimicrobial and insecticidal properties^66,67^. Glycosides are better known for their physiological effect on the cardiovascular system, with cardiac glycosides being the drug of choice for treating congestive heart failure^68^. Thus, the presence of these essential phyto-compounds in CNSW extracts signifies the importance of this wasted raw material.

The physico-chemical properties of CNSL are presented in Table 2, and were all determined using hexane extracted CNSL, because the quality and yield were better than the ethanolic and petroleum ether extracted-CNSLs respectively. The extracted CNSL was a reddish-brown viscous oil with a pH value of 3.2, probably due to a high concentration of anacardic acid and other phenolic compounds^8^. The moisture and ash content of the CNSW biomass expressed in percentages were 6.5% and 1.6 ± 0.1% respectively. These values were in line with 6.7% and 1.3% reported by Ref.^1^. The specific gravity, and refractive index values were 0.9561 ± 0.0002 (g/cm^3^) and 1.52 ± 0.001 respectively. These values were higher than 0.9118 (g/cm^3^) and 1.4325 reported by Ref.^39^, but lower than 1.686 and 0.9999 (g/cm^3^) reported by Refs.^1,69^ respectively.

**Table 2:** Physicochemical properties of CNSL from Mongu waste cashew nut shell.

Physicochemical properties	This study	Literature value
Colour	Dark Brown	Dark Brown (Idah et al.^7^)
Odour	Choke	Choke (Idah et al.^7^)
Nature	Viscous liquid	Viscous liquid (Akinhanmi et al.^1^)
Moisture (%)	6.50	6.7 (Akinhanmi et al.^1^)
Ash (%)	1.6 ± 0.1	1.3 (Akinhanmi et al.^1^)
Specific Gravity (30 °C)	0.9561 ± 0.0002	0.9995 (Mohammed^69^)
Refractive Index (20 °C)	1.52 ± 0.001	1.686 (Akinhanmi et al.^1^)
Viscosity (mPa-s ,24 °C)	104.6 ± 1.8	150–600 (Rodrigues et al.^11^)
Acid Value (mg KOH/g)	118.7 ± 9.2	141 and 112 (Achi and Myina^70^; Mahanwar and Kale^71^)
Free Fatty Acid (%)	60.1 ± 4.7	58 (Akinhanmi et al.^1^)
Saponification (mg KOH/g)	138.1 ± 3.2	161 (Achi and Myina^70^)
Iodine Value (g I_2_/100 g)	188.1 ± 2.3	177.7 (Achi and Myina^70^)
pH	3.2	3.0 (Achi and Myina^70^)

Table shows the physicochemical properties of the cashew nut shell liquid extracted from roasted shells which were obtained from Mongu District of the Western Province of Zambia. Results were expressed as percent means ± SEM and milligram KOH, g I_2_ and viscosity was in mPa s respectively.

The viscosity of CNSL in this work was 104.6 ± 1.8 mPa s. This value was lower than 160 mPa s and 410 mPa s reported by (Mohammed^69^) and (Rodrigues et al.^11^). The standard viscosity range for CNSL at 25 °C is 150–600 mPa s^72^. The reason for low viscosity in this work could be that the shells were roasted under uncontrolled conditions by the local cashew nut farmers during processing. However, CNSL with low viscosity presents an advantage, as it can be blended with diesel to form biodiesel for heavy engines^73^. Biodiesels with high viscosity values are characterised with poor fuel atomization, larger droplet size and spray jet penetration, leading to inefficient mixing of fuel and air in the combustion chambers^74^. The acid and free fatty acid values were 118.7 ± 9.2 (mg KOH/g) and 60.1 ± 4.7% respectively. The acid value in this work was higher than most literature values 12.1, 15.5, and 112 (mg KOH/g) reported by Ref.^1,71,75^, but lower than 141 mg KOH/g reported by Ref.^70^. A high acid value suggests that CNSL cannot be consumed by humans or directly applied on acid sensitive surfaces to avoid corrosion. Ingestion of oils with high acid value leads to human gastrointestinal discomfort, diarrhea and liver damage^76,77^. Cashew nut shell liquid with high acid value is first neutralized with alkaline bases before it is applied in paints, vanishes and others surface coating agents^70^. The saponification number and iodine values for this work were 138.1 ± 3.2 (mg KOH/g) and 188.1 ± 2.3 (gI_2_/100 g) respectively. The obtained saponification number was lower than 161 mg KOH/g reported by^7^. Saponification number depend on the amount of fatty acids present in a fat or oil sample. The higher the number, the higher the amount of fatty acids and vice versa. It is also used to determine the average molecular weight of fatty acid chains in fats/or oils^74^. Fats/ or oils with long fatty acids have low saponification values because they have fewer carboxylic group per unit mass of fat/oil, as compared to short chain fatty acids. Fatty acids with longer chains make good surfactants. Their surfactants have excellent detergent properties and they do not irritate the skin^78^. Iodine value indicates the unsaturation of fats/or oils^74^. The value 188.1 ± 2.3 (mgI_2_/100 g) obtained in this work was in line with and 177.7 (mg I_2_/100 g) reported by Ref.^7^. The higher the iodine value, the more unsaturated the fat/or oil sample is. Highly unsaturated oils or fats are good for paints and surface coating materials, as they dry faster and their conjugated double bonds help to slowdown the oxidation process of painted objects^1^. The difference in the composition and physicochemical properties of CNSL in this work and other literature sources may be due to variation in the species, climate and geography where cashew was grown as well as the operating conditions employed during analysis^79^.

### Batch adsorption of heavy metals (copper, lead, cadmium and zinc) onto CNS-AC

The cashew nut shell activated carbon (CNS-AC) was used to remove heavy metals (lead, cadmium, copper and zinc) from synthetic aqueous solutions. The batch adsorption was carried out at conditions of 1 g adsorbent dosage, 0.002 to 3 mg/L initial metal concentration, 30 mL of adsorbate solution, pH of 6.98, 30 min contact time and agitation speed of 250 rpm. The average percentage removal of Cu (II), Pb (II), Cd (II) and Zn (II) is shown in Table 3.

**Table 3:** Percentage removal of heavy metals by CNS-AC.

Adsorbent	% Adsorption			
	Cu	Pb	Cd	Zn
CNS	99.4 ± 0.5	95.4 ± 1.5	99.4 ± 0.2	99.5 ± 0.1

Table Shows the percentage heavy metal removal by 1 g activated carbon at room temperature and pressure.

### Batch adsorption of MB onto CNS-AC

Some of the factors that affects adsorption such as pH, temperature, contact time and concentration were considered in this study.

#### Effect of solution pH on MB adsorption onto CNS-AC

**Figure 2:**
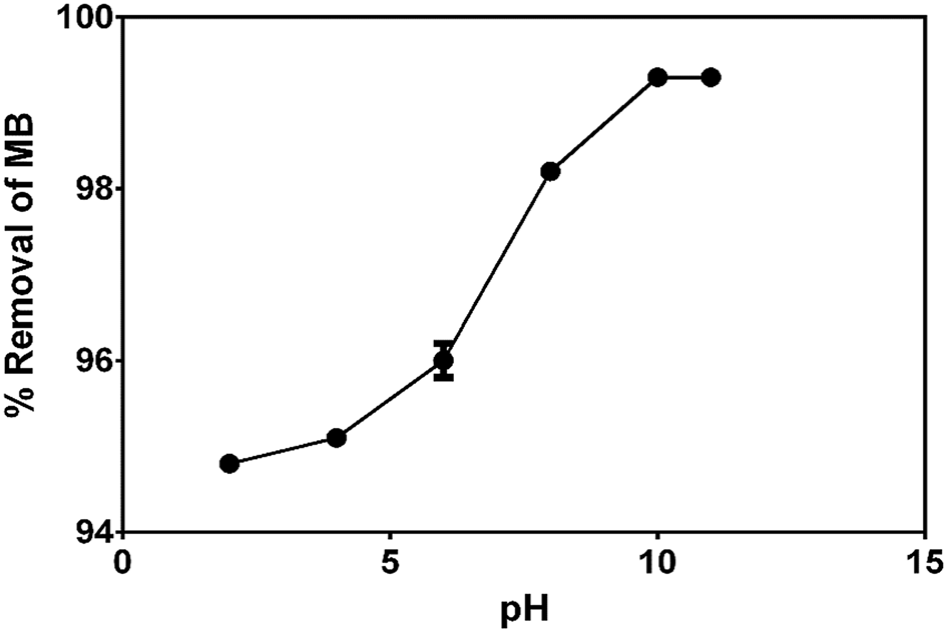
Adsorption efficiency of MB on CNS-AC at various pH values ranging from 2 to 11. The adsorbent dosage (S/L) was = 33.33 g/L, initial concentration of dye was 50 mg/L, total time was 160 min at 25 °C (298 K). Plots were generated in Graphpad Prism 9.1.0. Values were expressed as means ± SEM. (https://www.graphpad.com/).

The influence of initial pH value of the solution on the adsorption process of MB onto CNS-AC was carried out at 50 mg/L initial MB concentration, 298 K temperature and contact time of 160 min. The adsorption efficiency increases from 94.8 to 99.1% for an increase in pH from 2 to 11 (Fig. 2). The near sigmoidal adsorption pattern under different pH units agrees with other studies done on adsorbates for methylene blue removal from aqueous solutions ^80,81^. The adsorption of MB was highly favoured under basic compared to acidic conditions with the highest removal percentage of 99.1 at pH 10. Uptake of MB by CNS-AC was constant at pH 10 and 11 as shown in Fig. 2. The low adsorption efficiency of MB in acidic media could be attributed to high competition for adsorption sites between the excess hydrogen ions (H^+^ ions) in the solution and the cation groups on MB^80^.

#### Effect of initial dye concentration and contact time on adsorption of MB onto CNS-AC

**Figure 3:**
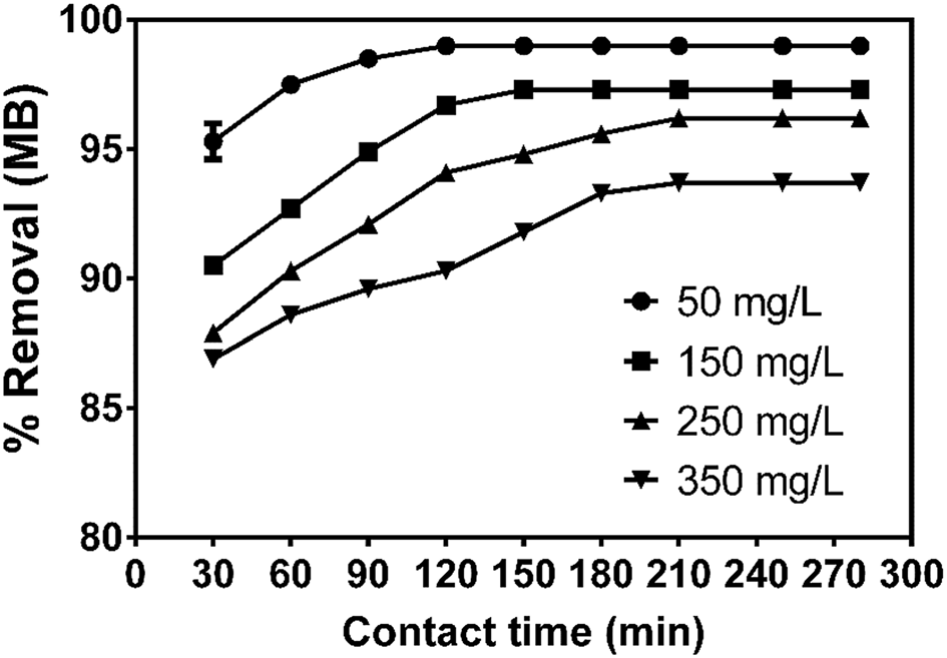
MB concentration ranged from 50 to 350 mg/L at an adsorbent dosage (S/L) of 33.33 g/L, pH 10 and contact time from 30 to 280 min at room temperature and pressure. Plots were generated in Graphpad Prism 9.1.0 version Values were expressed as means ± SEM (https://www.graphpad.com/).

The relationship between adsorption of MB and contact time was investigated to establish the rate of MB removal. Figure 3 shows the plot of removal percentage versus contact time for different MB concentrations ranging from 50 to 350 mg/L. The adsorption of MB increased with the increase in contact time until equilibrium was reached in about 120, 150, 210 and 250 min for an MB concentration of 50, 150, 250 and 350 mg/L respectively. Also, the percentage removal decreased from 99 to 93.74% for an increase in initial MB concentration from 50 to 350 mg/L. The reason for this behaviour can be attributed to the fact that, there are more active adsorption sites on the surface of the adsorbent compared to the total MB molecules in solution at lower concentrations, thus, more molecules interacts with the adsorbent and are removed from the solution^54,80^.

#### Adsorption isotherms

**Figure 4:**
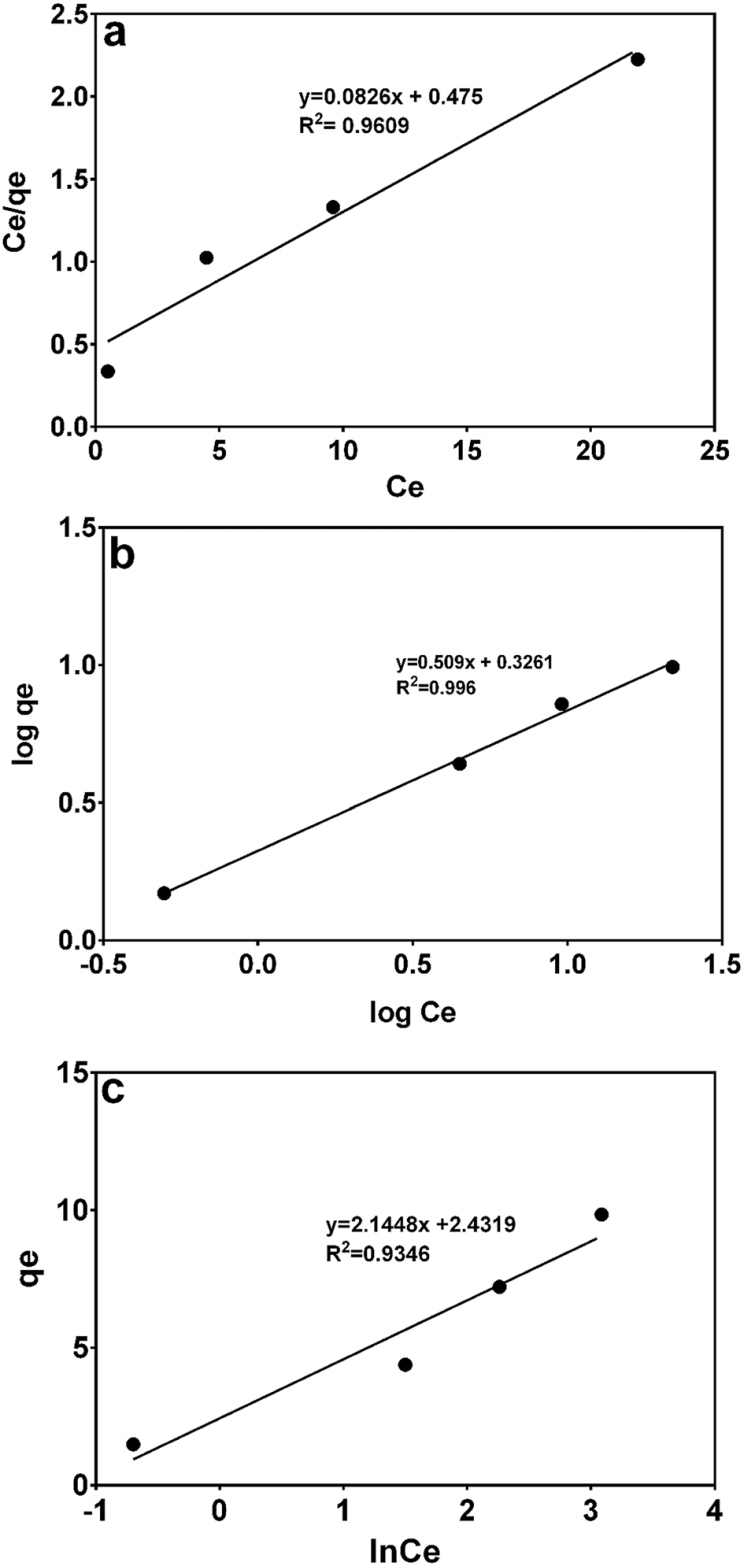
(**a**) Langmuir, (**b**) Freundlich, and (**c**) Temkin isotherm plots for adsorption of MB onto CNS-AC. The data was best fit to the Freundlich isotherm with a correlation coefficient of 0.996.

Analysis of isotherm models is significant in modelling and designing of the adsorption process as they show the distribution of the adsorbate molecules between the liquid phase and the solid phase when an equilibrium state is reached. In this study, Langmuir, Freundlich and Temkin isotherm models were considered. The isotherm constants and regression coefficients (R^2^) calculated from adsorption experiments are given in Table 4. The Freundlich isotherm model was suitable since regression coefficient (R^2^) was higher than that of the Langmuir and Temkin isotherm models as shown in Fig. 4. Thus, adsorption of MB onto CNS-AC fitted best the Freundlich isotherm model.

**Table 4:** Values of Langmuir, Freundlich and Temkin isotherm constants for adsorption of MB onto CNS-AC.

q_max_(mg/g)	Langmuir constants			Freundlich constants			Temkin constants		
	K_L_	R_L_	R^2^	K_F_	n	R^2^	b	K_T_	R^2^
12.1	0.174	0.016–0.103	0.9609	2.12	1.965	0.996	1155.15	3.108	0.9346

Table shows the three adsorption isotherms; Langmuir, Freundlich and Temkin with the Langmuir isotherm yielding 12.1 mg/g qmax and overall the data being best fit to the Freundlich isotherm with a correlation coefficient of 0.996.

#### Adsorption kinetic models

The adsorption kinetic models are important in evaluating the rate and kinetic behaviour of the adsorption process. The kinetic parameters provide substantial information in designing and modelling of the adsorption process. The kinetic of methylene blue (MB) adsorption onto CNS-AC was analysed using pseudo-first-order and pseudo-second-order kinetic models.

**Figure 5:**
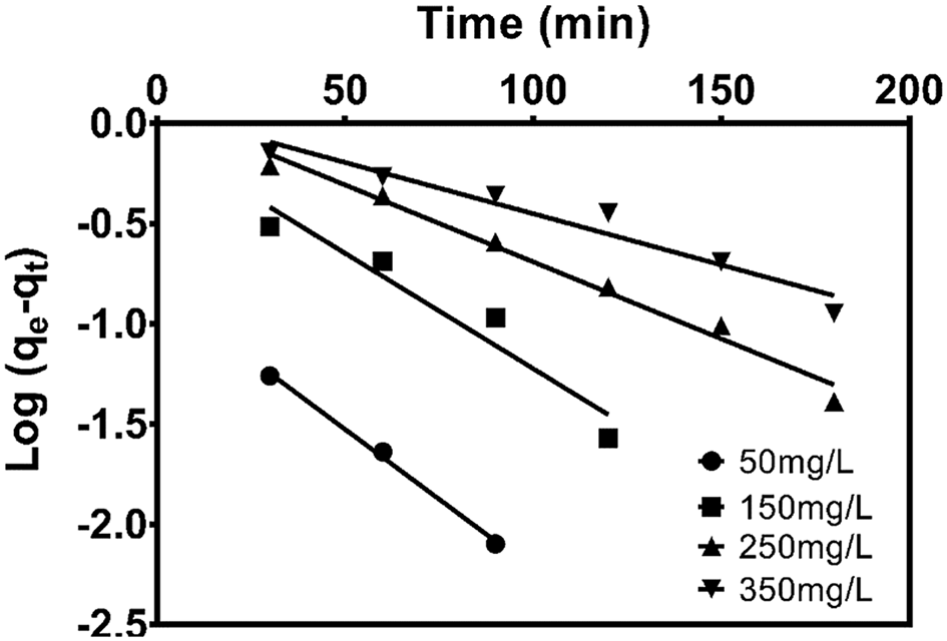
Pseudo-first-order kinetics of MB adsorption on CNS-AC at different initial MB concentration, pH 10, S/L = 33.33 g/L and T = 298 K.

A pseudo-first-order kinetic equation is given as;

7 $$\log \left( {q_{e} - q_{t} } \right) = logq_{e} - \frac{{K_{1} }}{2.303}t,$$

where; $q_{e}$ and $q_{t}$ (mg/g) are the amounts of methylene blue (MB) adsorbed at equilibrium and at time t (min), $K_{1}$(min^−1^) is the adsorption rate constant. The parameters $q_{e}$ and $K_{1}$ were determined from the intercept and slope of a plots of $\log \left( {q_{e} - q_{t} } \right)$ versus t as shown in Fig. 5. The parameters of pseudo-first-order kinetic are tabulated in Table 4.

**Figure 6:**
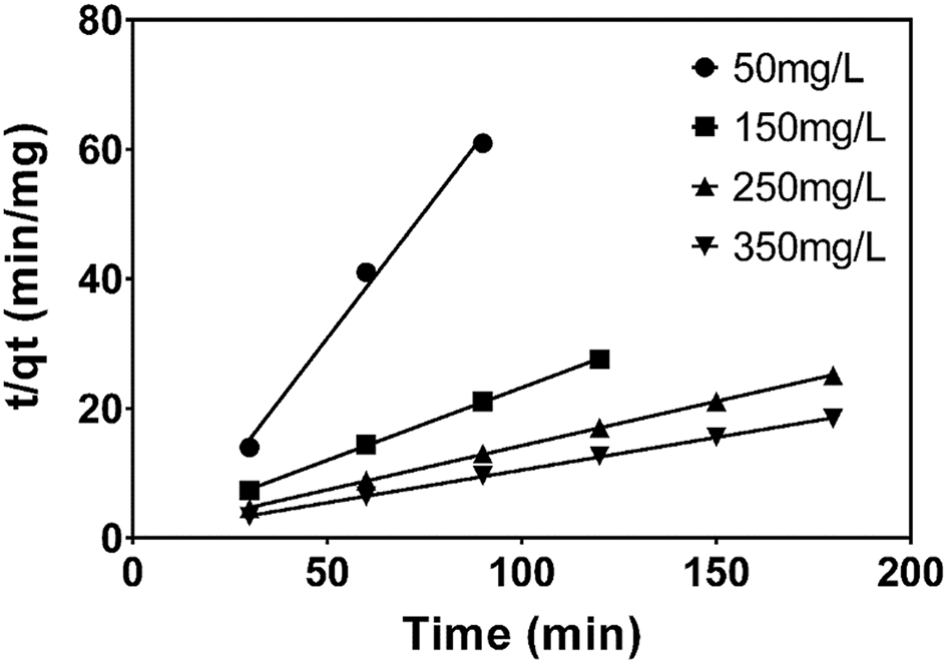
Pseudo-second-order kinetics of MB adsorption on CNS-AC at different initial MB concentrations, pH 10, S/L = 33.33 g/L and T = 298 K.

Pseudo-second-order kinetic model is expressed as;

8 $$\frac{t}{{q_{t} }} = \frac{1}{{K_{2} q_{e}^{2} }} + \frac{1}{{q_{e} }}t,$$

where; $K_{2}$ (g/mg/min) is second order adsorption rate constant, h (mg/g/min) is the initial adsorption rate. The parameters $q_{e}$ and $K_{2}$ were calculated from the slope and intercept of the plots of $\frac{t}{{q_{t} }}$ versus t as shown in Fig. 6.

Pseudo-first-order and pseudo-second-order kinetic parameters for different initial concentrations of methylene blue are tabulated in Table 5. The value of the correlation coefficient (R^2^) for pseudo-second order model is higher than the value of pseudo-first-order adsorption model. Furthermore, pseudo-second-order model has values of $q_{e}$_, cal_ which are close to $q_{e}$_, exp._ It can be concluded that the adsorption of methylene blue onto CNS-AC follows pseudo-second-order kinetic model. This implies that, the rate-limiting step is the surface adsorption that involves chemisorption. Thus, chemical adsorption likely occurs through the formation of a covalent bond.

**Table 5:** Pseudo-first-order and pseudo-second-order kinetic models for the adsorption of methylene blue (MB) onto CNS activated carbon.

Kinetic parameters	Initial concentration (mg/L)			
	50	150	250	350
q_e_, exp (mg/g)	1.485	4.378	7.212	9.843
**Pseudo-first-order kinetics**				
R^2^	0.9969	0.927	0.9829	0.9448
q_e_, cal (mg/g)	0.149	0.846	1.193	1.149
k_1_	0.032	0.026	0.018	0.012
**Pseudo-second-order kinetics**				
R^2^	0.9923	0.9997	0.9998	0.9995
q_e,_ cal (mg/g)	1.278	4.456	7.321	9.921
k_2_	0.074	0.066	0.031	0.026

Table shows the pseudo-first-order and pseudo-second-order kinetic models for the adsorption of methylene blue (MB) onto CNS activated carbon. The data best fits the pseudo-second order with a correlation coefficient of 0.9997, 0.9998 and 0.9995 at 150, 250 and 350 mg/g initial concentration. The values of q_e_ cal and q_e_ exp were correlated for the pseudo-second order kinetics model.

#### Effect of temperature and thermodynamic parameters

**Figure 7:**
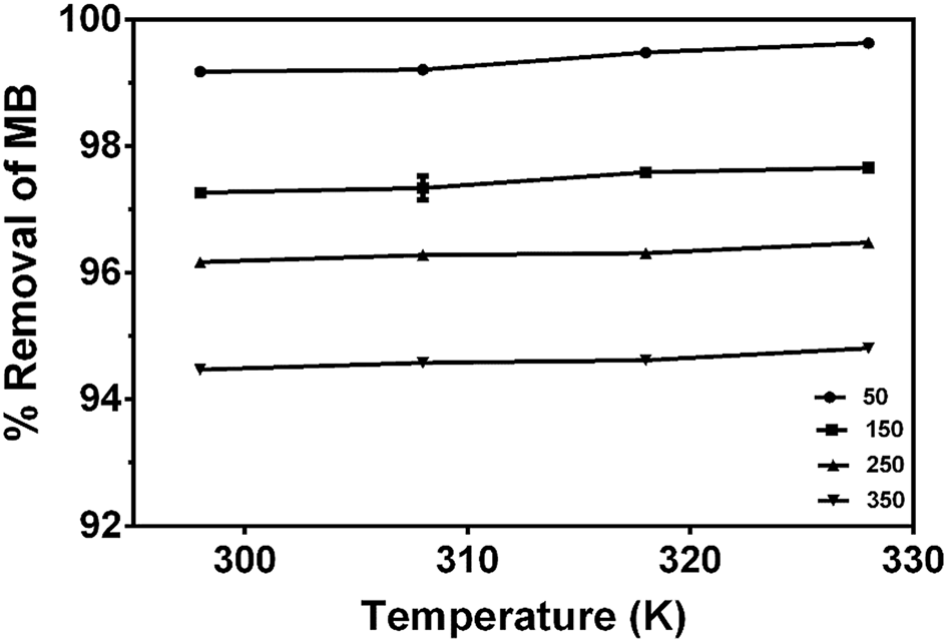
Effect of temperature on percentage MB removal by CNS-AC (adsorbent dosage S/L = 33.33 g/L, pH 10 and contact time = 120 min). Plots were generated in Graphpad Prism 9.1.0 version. Values were expressed as means ± SEM (https://www.graphpad.com/).

The influence of temperature on adsorption of MB using CNS-AC was investigated at different temperatures (298, 308, 318 and 328 K) and MB concentrations of 50, 150, 250 and 350 mg/L. Increasing the temperature from 298 to 328 K as shown in Fig. 7 increased the adsorption efficiency of MB for all the concentrations from 94.81 (350 mg/L) to 99.63 (50 mg/L). Thus, increase in temperature increases the thermal motion, chemical potential and solubility of MB molecules^82^, thereby, enhancing the interaction of MB molecules with the adsorbent. The maximum removal efficiency of MB by CNS-AC at 50, 150, 250 and 350 mg/L was found to be 99.63, 97.66, 96.48 and 94.81, respectively.

Thermodynamic study of adsorption process of MB onto CNS-AC to estimate the feasibility of the adsorption process was investigated. The Gibbs free energy change (Δ*G*°) values are useful in determining whether the process is spontaneous or not. A positive value of Δ*G*° means that the adsorption process is non-spontaneous and a negative value shows that the process is spontaneous. The enthalpy change (Δ*H*°) differentiates a physical adsorption process from a chemical adsorption process and provides information about the exothermic nature or endothermic nature of the adsorption process^83^. The entropy change (ΔS^o^) indicates the disorder of the solid/solution interface during the adsorption process.

The change in Gibbs free energy (Δ*G*°) was calculated using the following equations^54,84^;

9 $$\Delta G^\circ = - RTInK_{C} ,$$

10 $$K_{C} = \frac{{q_{e} }}{{C_{e} }}.$$

**Figure 8:**
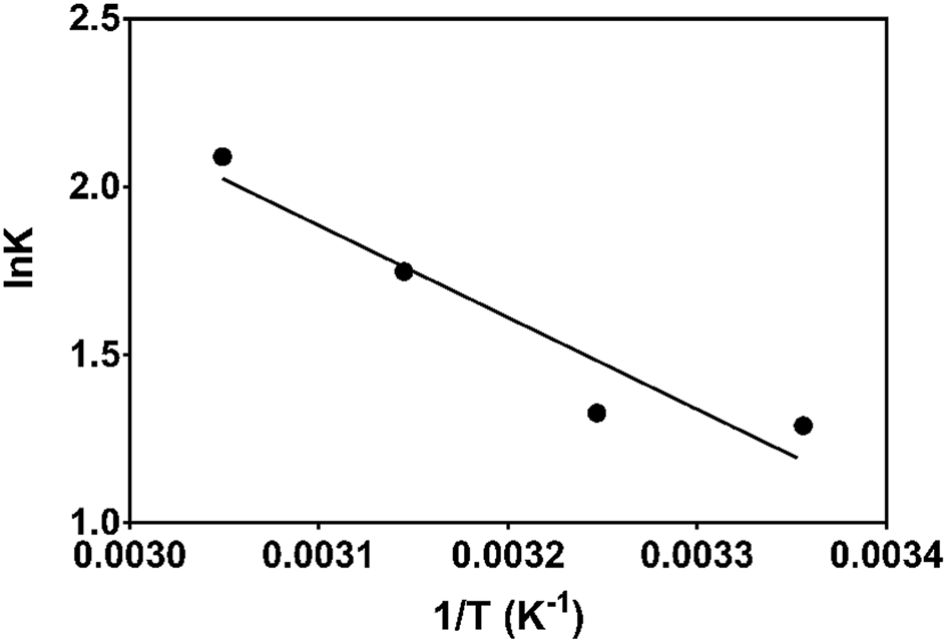
Plot for the thermodynamic parameters on the adsorption of MB by CNS-AC, 50 mg/L MB concentration and 33.33 mg/L adsorbate.

The enthalpy change (Δ*H*°) and entropy change (ΔS^o^) change was determined from the equation below^82^;

11 $$\Delta G^\circ = \Delta H^\circ - T\Delta S^\circ ,$$

where; T is the absolute temperature (K), R is the universal gas constant (8.314 J/mol/K), ∆G° (kJ/mol) is the Gibbs free energy change, ∆H° (kJ/mol) is the enthalpy change and ∆S° (kJ/mol/K) is the entropy change. The values of ∆S° and ∆H° (Table 6) are obtained from the slope and intercept of the plot of lnK versus 1/T (K^−1^) and are shown in Fig. 8.

**Table 6:** Thermodynamic parameters for MB adsorption on CNS-AC.

Temperature (K)	∆G° (kJ/mol)	∆H° (kJ/mol)	∆S° (kJ/mol/K)	R^2^
298	− 2.94	22.76	0.086	0.908
308	− 3.80			
318	− 4.66			
328	− 5.52			

Table shows the thermodynamic parameters for MB adsorption on activated carbon prepared from waste defatted cashew nut shell.

The negative values obtained for Gibbs free energy (∆G°) indicates the feasibility and spontaneity of the adsorption process. The positive value (22.76 kJ/mol) of enthalpy change (∆H°) indicates that the adsorption of MB by CNS-AC at different temperatures was endothermic. The value of the entropy change (ΔS°) was 0.086 kJ/mol/K implying that the randomness of solid/solution interface during adsorption process increased. These results were consistent with studies done by Ref.^54^.

## Conclusion

In this paper, we analyzed the potential use of cashew nut shell regarded as a waste in Zambia and indeed in many countries growing cashew. Solvent extraction and synthesis of activated carbon experiments were done to evaluate the potential value of cashew nut as a source of both chemical feedstock and activated carbon for use as a low cost filtration system matrix. Best yields of CNSL were achieved by hexane (38.2 ± 0.4%). Physicochemical results showed that CNSL has high potential as an intermediate in the synthesis of paints, varnishes, dyeing-stuff, binders, lubricants, nanotechnology^73^ and the presence of bioactive compounds such as alkaloids, steroids, terpernoids, polyphenols, saponins and glycosides indicates that CNSW can be a cheap source for pharmaceutical compounds^59,85^. The iodine ( 188.1 ± 2.3 gI_2_/100 g) and saponification (138.1 ± 3.2 mg KOH/g) values indicated that CNSL was a drying oil making it suitable for resins, surface coating materials^17,86^ and soap making respectively. The adsorption of heavy metals and MB onto CNS-AC has been studied. The average percentage removal of Cu (II), Pb (II), Cd (II) and Zn (II) was 99.4, 95.4, 99.5, 98.4%, and the removal efficiency of MB at 50, 150, 250 and 350 mg/L was 99.63, 97.66, 96.48 and 94.81, respectively. The study showed that increasing the initial pH, temperature and contact time increased the adsorption of MB onto CNS-AC and a decrease in initial MB concentration increased percentage removal of MB. Equilibrium data were fitted to Langmuir, Freundlich and Temkin isotherms models and the equilibrium data were best described by the Freundlich isotherm model. The maximum monolayer adsorption capacity was 12.1 mg/g. The kinetics of the adsorption process conformed to pseudo-second-order model and the negative value of the Gibbs free energy (∆G°) and positive value of enthalpy change (∆H°) indicates that the adsorption process was endothermic and spontaneous. This paper therefore provided useful information thatcashew nut shell can be be used as a source for CNS-AC, a suitable adsorbent for removal of heavy metals and organic soluble matter from water as modeled by Cd, Cu, Pb, Zn and methylene blue dye^30,80,82^ removal respectively. The waste shells on one hand are also as a source of cardanol, cardol and other phenolic compounds useful in the chemical industry^73,87,88^.

## Abbreviations

AC: Activated carbon
CIDP: Cashew Infrastructure Development Project
CNS: Cashew nut shell
CNS-AC: Cashew nut shell-activated carbon
CNSL: Cashew nut shell liquid
CNSW: Cashew nut shell waste
DRGS: Directorate of Research and Graduate Studies
FFA: Free fatty acid
GRZ: Government of the Republic of Zambia
MB: Methylene Blue
MIN: Minutes
NASREC: Natural and applied sciences research ethics committee
RPM: Revolutions per minute
SEM: Standard error of the mean
UNZA: University of Zambia
